# 

**Published:** 1893-06

**Authors:** 


					﻿POFVX-AIR FALLACIES.
By JAMES HILLS, M. D.
io cts. a copy.
JUNE, 1893. !
$1.00 per year.
HALES
eJ0VKN/\L‘J
HEALTH
ESTABLISHED 1854
Uouxl:
n/H-6,
OFFICE, 23 PARK ROW.
Cnoa-Qn/'nnrl.Plaaa Xf uttor nt th A Pnat.Oftlnp XIaw Vnrlr
				

